# COVID-19 and the Brain: Acute Encephalitis as a Clinical Manifestation

**DOI:** 10.7759/cureus.10784

**Published:** 2020-10-03

**Authors:** Asim Haider, Ayesha Siddiqa, Nisha Ali, Manjeet Dhallu

**Affiliations:** 1 Internal Medicine, BronxCare Health System, Bronx, USA; 2 Neurology, BronxCare Health System, Bronx, USA

**Keywords:** coronavirus disease 2019, rituximab, viral encephalitis, acute encephalopathy

## Abstract

Central nervous system (CNS) viral infections result in the clinical syndromes of aseptic meningitis or encephalitis. Although the primary target of coronavirus disease 2019 (COVID-19) is the respiratory system, it is increasingly being recognized as a neuropathogen. The hallmark clinical feature is altered mental status, ranging from mild confusion to deep coma. Most patients with encephalopathy or encephalitis are critically ill. We present a case of COVID-19-related encephalitis who presented with acute delirium and new-onset seizures. The patient responded well to treatment with intravenous immunoglobulins and rituximab.

## Introduction

Coronavirus disease 2019 (COVID-19) is a disease with a significantly broad spectrum of presentation and clinical syndromes. This novel infectious disease has been associated with acute respiratory distress syndrome (ARDS), thromboembolic syndrome, severe metabolic syndromes, severe acute tubular necrosis, electrolyte abnormalities, neurologic syndromes, and cardiac events, including myocarditis and arrhythmias [1-3]. Beijing Ditan reported the first case of viral encephalitis associated with COVID-19 in March 2020. The researchers confirmed the presence of severe acute respiratory syndrome coronavirus 2 (SARS-CoV-2) in the cerebrospinal fluid (CSF) by genome sequencing [4]. Since then, clinicians and researchers worldwide have been observing more and more neurological manifestations of COVID-19.

## Case presentation

A 66-year-old male, with a medical history of benign prostatic hypertrophy, fatty liver disease, and hypertension, admitted to the hospital after experiencing multiple new-onset seizures followed by persistent confusion. As per the family, he experienced intermittent confusion and bizarre behavior like staring at the walls for two days before the onset of seizures. He was seen in his primary care provider's clinic four days before the onset of seizures and was in the usual state of health at that time. Initial vital signs were: heart rate 90 beats per minute, respiratory rate 16 times per minute, temperature 98.4°F, oxygen saturation 98% on room air, and blood pressure 131/79 mmHg. On physical examination, the patient was confused, not oriented to time, place, or person, and could not follow commands. However, he did not have any focal neurological deficits or neck rigidity. Laboratory tests, including human immunodeficiency virus (HIV) antibodies, rapid plasma reagin (RPR), thyroid-stimulating hormone (TSH), vitamin B 12, and urine toxicology, were unremarkable. Computed tomography (CT) scan of the head and CT angiogram of the brain and neck were negative. Septic workup, including blood and urine cultures, were also negative. Due to a persistent state of confusion, he underwent a lumbar puncture and was started empirically on antibiotics (vancomycin, acyclovir, ceftriaxone, and ampicillin) for meningitis treatment. He also received phenytoin 100 mg three times per day for seizure prophylaxis. Lumbar puncture showed an opening pressure of 15 cm of H_2_O, cerebrospinal fluid (CSF) glucose level of 86 mg/dl, protein level of 77 mg/dl, and white blood cell (WBC) count of 3/mm^3^. CSF studies were negative for cultures (both bacterial and viral), cryptococcal antigen, and herpes simplex virus polymerase chain reaction (PCR) test (Table 1).

**Table 1:** Cerebrospinal fluid analysis PCR: polymerase chain reaction; VDRL: venereal disease research laboratory

Spinal fluid	Lab values	Reference range
Color	Colorless	Colorless
White blood cell count (mm^3^)	3	0-5
Red blood cells (mm^3^)	299	0
Glucose (mg/dl)	86	40-70
Protein (mg/dl)	77	15-45
Opening pressure (cmH_2_O)	15	5-20
Gram stain	Negative	Negative
Bacterial antigen	Negative	Negative
Herpes simplex virus 1 and 2 PCR	Negative	Negative
Cytomegalovirus PCR	Negative	Negative
VDRL	Nonreactive	Nonreactive

**Figure 1:**
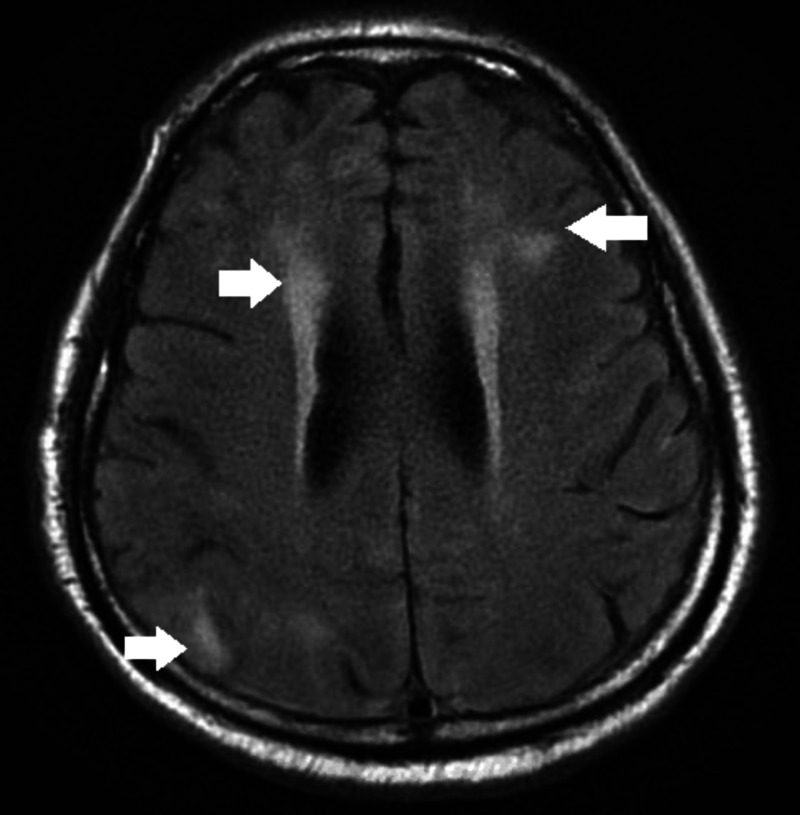
Magnetic resonance imaging (MRI) brain without contrast showing lateral periventricular and right parieto-occipital bright signals concerning for cerebritis

The patient was noted to have a positive PCR assay for SARS-CoV-2 in the nasopharyngeal swab. Autoimmune workup, including anti-N-methyl D-aspartate (NMDA) receptor antibodies, anti-Ro antibodies, anti-La antibodies, antineutrophil cytoplasmic antibodies (ANCA) antibodies, and anti-Hu antibodies, were negative. Blood tests, including Lyme antibodies by Western blot and varicella-zoster virus (VZV) PCR, were negative. Initial electroencephalography (EEG) demonstrated the right temporal epileptiform activity. Repeat EEG showed global cerebral dysfunction and severe toxic metabolic encephalopathy. Magnetic resonance imaging (MRI) brain without contrast showed small acute/subacute lacunar infarcts and a patchy area of T2 bright signals in the cortical and periventricular regions, concerning for cerebritis (Figure 1).

Due to suspicion of COVID-19-related encephalitis, the patient received two doses of tocilizumab (400 mg each) followed by intravenous (IV) immunoglobulin (1 g/kg) for five days. The patient's mental status did not improve even after completing the treatment with tocilizumab and IV immunoglobulins. The decision was made to start the patient on rituximab. The patient received one dose of rituximab (one gram) with significant improvement in mental status. He became more calm and co-operative afterward. He was discharged to a nursing home. Two months post-discharge, the patient was followed up and his mental status was much better; he was alert, oriented, and able to take care of his daily activities.

## Discussion

Although coronavirus's primary target is the respiratory tract, it is known to have neuroinvasive properties. However, the evidence on the central nervous system (CNS) involvement and neurological manifestations of COVID-19 is scarce and of low quality. A study that specifically investigated this issue documented that 36% of the hospitalized patients with a confirmed diagnosis of an acute respiratory syndrome from COVID-19 infection had some neurological manifestations. Neurological symptoms usually fall into one of three categories: CNS symptoms or diseases (headache, dizziness, impaired consciousness, ataxia, acute cerebrovascular disease, and epilepsy), peripheral nervous system (PNS) symptoms (hypogeusia, hyposmia, hypopsia, and neuralgia), and skeletal muscular symptoms [5]. Patients with severe symptoms are more likely to develop neurological symptoms than patients with mild or moderate disease [3].

The exact mechanism by which COVID-19 infects CNS is not well-understood due to a lack of experimental data, but it is considered a mutation of the Middle East respiratory syndrome (MERS) virus and severe acute respiratory syndrome (SARS) virus [3]. The target receptor for these coronaviruses is the angiotensin-converting enzyme-2 (ACE 2) receptor. After attachment and internalization, viral ribonucleic acid (RNA) is released into the cytoplasm, subsequently leading to translation and replication [6]. The ACE 2 receptor is also found in the glial cells of the brain and spinal cord tissues. There are at least three proposed mechanisms through which coronaviruses can enter the CNS: (a) retrograde transfer from the olfactory epithelium to the brain via cribriform plate, (b) damage to the blood-brain barrier during the viremia phase, and (c) transfer from peripheral nerve terminal to CNS via synapse connected route [6]. Following CNS invasion, neurological damage can occur via the following mechanisms: (a) immune-mediated damage in the setting of cytokine storm and (b) neuronal damage in the setting of significant hypoxia due to severe pneumonia and acute respiratory distress syndrome (ARDS) [7].

The diagnosis of COVID-19-related encephalitis can be extremely challenging, as the definitive diagnosis of viral encephalitis largely depends on virus isolation from CSF; this is difficult for COVID-19 because SARS-CoV-2 dissemination is transient and its CSF titer may be extremely low. Most of the patients of COVID-19, who had encephalopathy and underwent EEG, showed nonspecific findings [8]. Two case series involving CSF analysis data from 12 patients reported that the CSF had no white blood cells and the PCR assay for SARS-CoV-2 was negative in all the patients [9,10]. A spectrum of MRI findings has been described in patients with COVID-19-related encephalopathy, including leptomeningeal enhancement, ischemic strokes, and cortical fluid-attenuated inversion recovery (FLAIR) signals [9]. Isolated white matter microhemorrhages have also been described in patients with severe COVID-19-associated ARDS [11]. 

The treatment of COVID-19-related encephalitis is mainly supportive. A variety of treatments, including high-dose IV steroids, IV immunoglobulin, and immunomodulators (e.g., rituximab), have been tried in various cases, with somewhat limited outcomes [12]. Neurological dysfunction may persist in many cases after the symptoms of acute illness have been resolved. In a case series, one-third of such patients were cognitively impaired at discharge [10]. In our clinical experience, the patient is slowly improving to his baseline mental status.

## Conclusions

COVID-19 is primarily a respiratory pathogen, but there is increasing evidence that it infects both the CNS and PNS. The absence of any specific findings on EEG, CT head, MRI head, and CSF analysis essentially makes it a disease of exclusion. The neurological manifestations of this virus are a rapidly evolving area but current evidence is limited. Therefore, it is essential to collect reliable data on short- and long-term neurological manifestations worldwide.
